# A Novel Polyphosphate‐Degrading Enzyme Confers Growth on Exogenous Polyphosphate in the Archaeon *Haloferax volcanii*

**DOI:** 10.1111/1462-2920.70340

**Published:** 2026-06-30

**Authors:** Jack W. F. Nicholls, Vincent O'Flaherty, Timothy M. Lenton, John W. McGrath, Jason P. Chin

**Affiliations:** ^1^ School of Biological Sciences Queen's University Belfast Belfast UK; ^2^ School of Biological and Chemical Sciences and Ryan Institute University of Galway Galway Ireland; ^3^ Global Systems Institute University of Exeter Exeter UK

**Keywords:** Archaea, biogeochemical cycles, exopolyphosphatase, haloarchaea, phosphorus cycling, polyphosphate

## Abstract

Polyphosphate (polyP) is an inorganic biopolymer of orthophosphate (Pi) that plays roles in many central biochemical processes in microorganisms, including acting as a cellular reservoir of phosphorus. The microbial metabolism of polyP is considered limited to its intracellular cycling by kinases and exopolyphosphatases. However, little is known about polyP metabolism in the Archaea. Notably, many haloarchaea, including *Haloferax volcanii* , encode homologues of bacterial polyP‐synthesising genes, but lack the corresponding genes for its catabolism. Here, we show that *H. volcanii* possesses a novel polyP‐degrading enzyme with characteristics distinguishing it from currently known enzymes. This activity also enabled *H. volcanii* to grow on exogenous polyP as its sole source of phosphorus. Activity was localised to a discrete protein band by native‐PAGE zymography and enzyme assays of cell‐free extracts from cells grown on polyP revealed the enzyme was halophilic, required Mn^2+^ or Co^2+^ and released Pi from polyP. No activity was observed in the extracellular medium of cells grown on polyP, suggesting the enzyme is either membrane‐bound or intracellular. These findings thus broaden our understanding of the potential ecological roles of polyP in the biogeochemical cycling of phosphorus, and our understanding of the enzymes and pathways involved in archaeal polyP metabolism.

## Graphical abstract

A novel polyP‐degrading enzyme with an activity differing from those currently known was found in *Haloferax volcanii* , conferring the ability for this microbe to grow on extracellular polyP. These findings further our understanding of the potential ecological roles of polyP and how archaea metabolise this essential biopolymer.

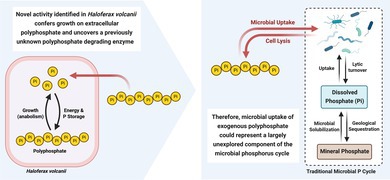

## 1 Introduction

Polyphosphate (polyP) is an inorganic biopolymer of three to hundreds of orthophosphate (Pi) residues bound by phosphoanhydride bonds and produced by cells across all domains of life. In microorganisms, polyP serves as a dynamic intracellular reservoir of phosphorus and energy (Seufferheld et al. 2011; Lander et al. 2016), but is also involved in many other key cellular processes such as stress responses, biofilm formation, virulence and gene expression (Rao et al. 2009; Muller et al. 2019).

**FIGURE 1:**
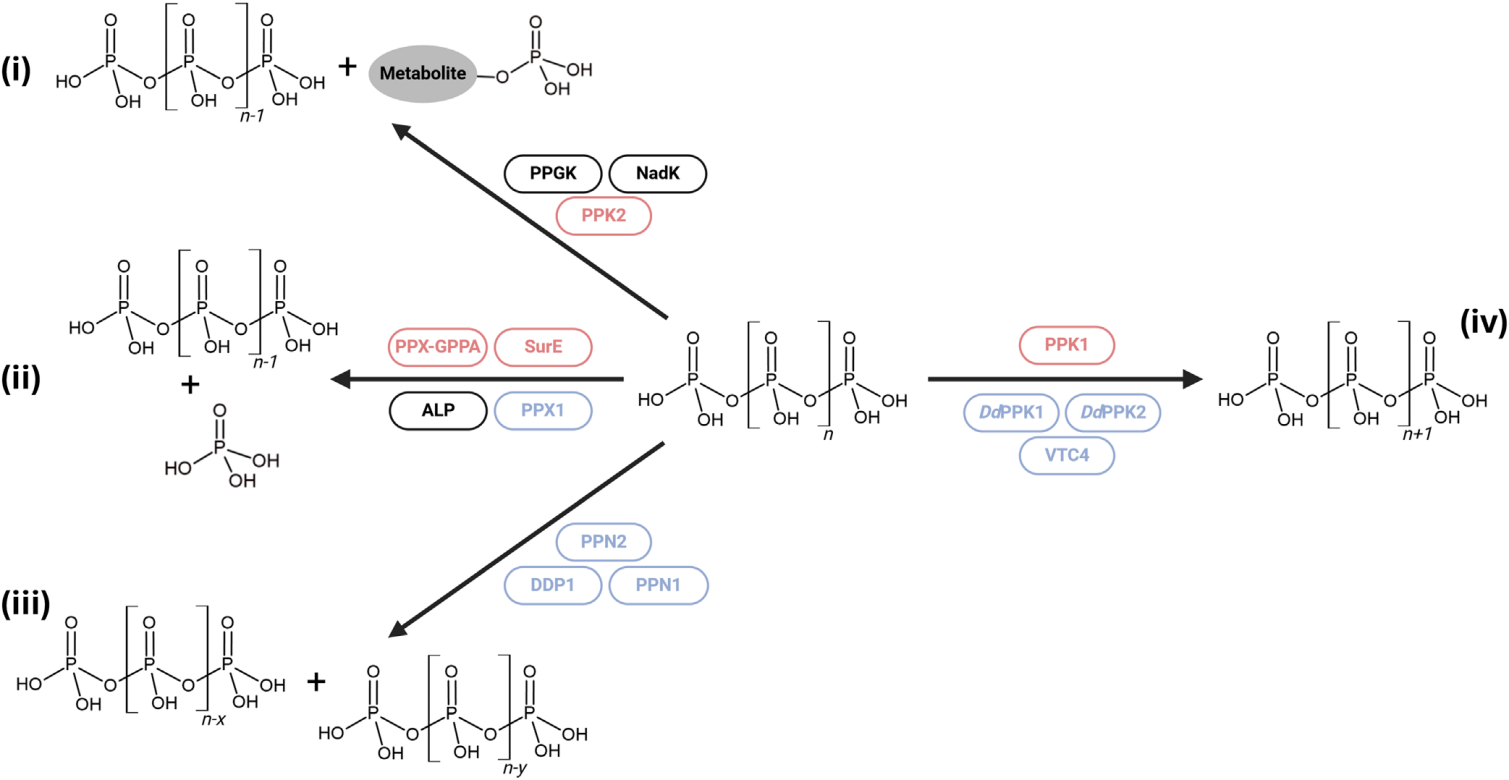
The major microbial enzymes that are known to degrade or synthesise polyphosphate. The three differing degradation activities, endopolyphosphatase (i), exopolyphosphatase (ii) and phosphoryl‐transfer (iii), are illustrated, along with its synthesis by various kinases (iv). Enzymes from microbial eukaryotes (blue) and those from prokaryotes (red) are shown, along with those found in both prokaryotic and eukaryotic microorganisms (black).

The numerous biochemical processes involving polyP make understanding the regulation of its metabolism crucial. In microorganisms, polyP is generally considered to be metabolised solely intracellularly by a variety of (de)phosphorylating enzymes (summarised in Figure 1 and reviewed by Albi and Serrano 2016; Gerasimaitė and Mayer 2016; Muller et al. 2019). In bacteria, synthesis is carried out by polyphosphate kinase 1 (PPK1; EC 2.7.4.1) and degradation by exopolyphosphatase (PPX; EC 3.6.1.11). Some bacteria also encode a polyphosphate kinase 2 (PPK2; EC 2.7.4.34) enzyme that reversibly synthesises polyP, while microbial eukarya contain different enzymes including the vacuolar transport chaperone complex subunit 4 (VTC4; EC 2.7.4.1) and endopolyphosphatases (PPNs; EC 3.6.1.10).

Despite extensive research in eukarya and some bacteria, polyP metabolism in the archaea remains largely unexplored. Investigation in two *Sulfolobus* species has revealed functional archaeal polyP‐metabolizing enzymes—one a homologue of the bacterial PPX and the other a distinct PPK‐type enzyme (Cardona et al. 2002; Höfmann et al. 2026). Bioinformatic studies have also highlighted that bacterial polyP‐cycling gene homologues are distributed across some archaeal lineages (Orell et al. 2012; Wang et al. 2018; Paula et al. 2019). For instance, some methanogens appear to encode homologues of the full canonical pathway of bacterial polyP metabolism (PPK1, PPK2 and PPX), while species from the ‘TACK’ superphyla contain selected PPX homologues. However, notably, many archaeal species lack the genes for any previously characterised polyP‐metabolizing enzymes or only encode incomplete polyP cycling pathways—highlighting the potential for polyP to be metabolised by hitherto unknown enzymes in these organisms.

Previous work from our group and others has revealed the widespread presence of homologues of the bacterial PPK1 enzyme in many haloarchaea, suggesting a common ability to synthesise polyP via this route. Yet most of these PPK1‐encoding haloarchaea did not encode any canonical bacterial or eukaryotic enzymes for the degradation of polyP (Orell et al. 2012; Paula et al. 2019; Wang et al. 2019). Therefore, alternative unknown mechanisms for polyP utilisation likely exist in these haloarchaeal organisms, including in the model haloarchaeon *Haloferax volcanii*, warranting further investigation of their polyP metabolism.

Beyond its intracellular roles, polyP may also exist in dissolved, extracellular forms in natural environments. The potential ecological roles of polyP remain poorly understood in part because polyP is primarily considered as an intracellular biomolecule but also because few studies have attempted to determine dissolved polyP concentrations or to distinguish between intracellular and extracellular polyP. While abiotic sources of polyP are thought to be extremely limited (Keefe and Miller 1995), the constant lytic turnover of microorganisms (some of which accumulate substantial intracellular polyP) suggests that dissolved polyP could be prevalent in the environment. Thus, if polyP is bioavailable to microorganisms, it could act as an important source of phosphorus and play unrecognised roles in the biogeochemical phosphorus cycle (Diaz et al. 2008).

Indeed, limited observations in some marine bacteria and algae shows that polyP can be consumed as an extracellular source of phosphorus (Diaz et al. 2018, 2019; Adams et al. 2022; Filella et al. 2022). However, the mechanisms underlying exogenous polyP consumption in these organisms, including how polyP is accessed and if it is transported into the cells, remains unknown. Given that the cellular mechanisms underpinning environmental polyP utilisation remain unexplored, our understanding of the diversity of microorganisms capable of exploiting this potentially valuable reservoir of phosphorus remains limited. Haloarchaea inhabit hypersaline environments characterised by high rates of microbial turnover and nutrient limitation, conditions under which dissolved organic phosphorus compounds, including polyP, may represent an important and underexplored phosphorus source in these environments.

Here, we set out to investigate whether haloarchaea harbour novel pathways for the degradation of polyP. Additionally, the capacity for haloarchaea to cycle polyP intracellularly or extracellularly was also explored. We now report that the haloarchaeon *H. volcanii* harbours a novel polyP‐degrading activity that enables growth on extracellular polyP as the sole phosphorus source. This activity was attributed to an enzyme that exhibits distinct biochemical properties, including halophily, metal dependence and an estimated approximate molecular mass that differentiate it from known microbial polyP‐degrading enzymes. Our findings therefore reveal a novel enzymatic route for the degradation of polyP in the haloarchaea and highlight the potential bioavailability of extracellular polyP in the environment, expanding our understanding of archaeal polyP metabolism and the potential ecological roles of polyP in the biogeochemical cycling of phosphorus.

## 2 Experimental Procedures

### 2.1 Glassware and Reagents

All culture vessels and glassware were cleaned with phosphate‐free detergent (2% Decon 90) to ensure no external phosphorus contamination. Unless otherwise stated, all chemicals were obtained from Sigma Aldrich, UK.

### 2.2 General Microbial Culturing

The *Haloferax volcanii* strain H295, a derivative of the wild‐type DS2 (Delmas et al. 2009), was provided by Thorsten Allers at the University of Nottingham. Optimal growth was achieved in a rich Payne's medium consisting of (per litre): sodium chloride (150.0 g), magnesium chloride heptahydrate (20.0 g), yeast extract (10.0 g, Fisher Scientific, USA), casamino acids (7.5 g, MP Biomedicals Inc., USA), trisodium citrate (3.0 g), potassium chloride (2.0 g), iron sulfate tetrahydrate (0.023 g) and manganese chloride tetrahydrate (0.023 g). The medium was adjusted to pH 7.4 and autoclaved at 121°C for 15 min prior to use. Cultures were then routinely incubated in flasks at 45°C and 150 rpm.

### 2.3 Growth Experiments

Cells of *H. volcanii* were first starved of phosphorus before testing for growth on polyP. For this, cultures were first grown for 72 h in Payne's medium, propagated into a phosphorus‐free minimal medium and incubated for 72 h, and then propagated into minimal media containing either 1 mM Pi, no phosphorus source, or 1 mM of polyP type 45 (polyP45, Sigma Aldrich).

Phosphorus‐free minimal medium contained (per litre): sodium chloride (150.0 g), magnesium sulfate pentahydrate (41.0 g), HEPES sodium salt (6.5 g) and potassium chloride (2.0 g). The pH was adjusted to 7.4 and the medium autoclaved at 121°C for 15 min. Filter‐sterilised 1000× trace element (Krieg 1981) and trace vitamin (Difco Laboratories 1953) solutions were added to autoclaved minimal medium to a final concentration of 1X. The following filter‐sterilised stocks were also added post‐autoclave: uracil (50 μg mL^−1^), glycerol (1 g L^−1^), ammonium chloride (5 mM) and either potassium dihydrogen phosphate (1 mM), polyP45 (1 mM) or phosphorus was omitted entirely.

During propagation, cells were washed twice by centrifugation at 10000 ×*g* for 10 min with resuspension in phosphorus‐free minimal medium. Fresh medium was inoculated with cell suspension to achieve a starting O.D. 600 nm of ~0.05. Growth experiments were performed in triplicate 20 mL cultures over a period of 72‐h, with incubation at 45°C and 150 rpm.

### 2.4 Culture Measurements

Cell growth was routinely measured by monitoring optical density at 600 nm (O.D. 600 nm) with a FLUOstar Omega Plate Reader (BMG LABTECH, Germany). Total protein was measured using the method of Binks et al. (1996). Briefly, 0.2–1 mL of culture was centrifuged at 18000 ×*g* for 15 min and the cell pellets lysed with 1:1 vol/vol (original sample volume) 0.5 M trichloroacetic acid for 30 min at room temperature (RT). Lysate was centrifuged at 18000 ×*g* for 15 min, the pellet resuspended in 1 mL of 0.66 M NaOH and incubated at 30°C overnight. Protein quantification was performed via the Bradford assay (Pierce Bradford Protein Assay kit, Thermo Scientific), as per manufacturer's instructions.

Dissolved Pi concentration in culture supernatant was measured with the BIOMOL green assay (Enzo Life Sciences Inc.) as per manufacturer's instructions. Samples were prepared by removing 0.2 mL culture, centrifuging at 18000 ×*g* for 15 min and storing the supernatant at 4°C until required.

### 2.5 Cell‐Free Extract Preparation

Phosphorus‐starved cultures of *H. volcanii* were prepared as above but grown in 200–400 mL of phosphorus‐free minimal medium, then propagated into 1 L of minimal media containing either 1 mM polyP45, 1 mM Pi or no phosphorus. Cells were then pelleted by centrifugation at 10000 ×*g* for 10 min at 4°C. The cell pellets were then resuspended in 20 mL of filter‐sterilised cell‐free extract buffer (2 M NaCl, 50 mM Tris–HCl pH 7.4 buffer and 2 mM EDTA in dH_2_O) and sonicated on ice with a Soniprep 150 Plus sonicator (MSE UK Ltd., UK). Sonication was by 10 bursts of 20 s at 10 μm amplitude with 20 s rests. Cell lysate was centrifuged at 18000 ×*g* for 15 min at 4°C, the supernatant syringe filtered (0.22 μm), and the resulting cell‐free extracts were stored in 20% v/v glycerol at −20°C. Protein quantification was via the Bradford assay (Pierce Bradford Protein Assay kit, Thermo Scientific), as per manufacturer's instructions.

### 2.6 Enzyme Activity Assays

PolyP‐degrading activity in cell‐free extracts of *H. volcanii* was determined with enzyme assays, in triplicate and with 1 mL or 100 μL reaction volumes. Unless otherwise stated, reactions consisted of 0.8–1.0 mg mL^−1^ of cell‐free protein extract (from *H. volcanii* cells grown on 1 mM PolyP45), 50 mM pH 7.4 HEPES buffer, 1 M KCl, 1.25 M NaCl, 1 mM MnCl_2_, 10 mM of polyP45 or other substrate when stated, in dH_2_O. The substrate was added last to initiate the reactions, which were then incubated at 37°C for 16 h in a ThermoMixer (Eppendorf, Germany), with constant mixing at 800 rpm. In all assays, triplicate substrate‐free and protein‐free control reactions were performed alongside the treatments.

PolyP degradation in assays was monitored by measuring the Pi concentration at the start and end of the reactions using the BIOMOL green assay (Enzo Life Sciences Inc.) as per manufacturer's instructions. For this, 20 μL samples were taken at *T*_0_ and *T*_end_ (and sometimes intermediate time points) and the BIOMOL assay was performed on samples immediately. Enzyme activity was calculated as nanomoles of Pi released from the substrate per mg of protein per minute.

For the optimal pH assay, 50 mM of Tris–HCl, HEPES or CAPS buffer at differing pH's were used. For salt requirement assays, KCl and/or NaCl were omitted. For metal cofactor requirement assays, MgSO_4_, CoSO_4_ and ZnSO_4_ were tested at 1 mM concentration. For substrate assays, varying polyP polymers (tripolyP; polyP3, polyP type 25; polyP25, polyP45 and polyP type 100; polyP100, Sigma Aldrich, UK) and nucleoside phosphates (ATP, ADP and AMP) were tested at 10 mM. Protein concentration and incubation temperature were also varied in their respective assays. Protein extract was also treated with 20 mg mL^−1^ Proteinase K (Thermo Scientific Inc., USA) or 10 mM EDTA during inhibition assays.

To measure extracellular enzyme activity, cultures of *H. volcanii* grown on 1 mM PolyP45 as the sole phosphorus source were prepared to a final volume of 300 mL. The whole culture volumes were then centrifuged at 10000 ×g for 10 min at 4°C and the supernatant passed through a 0.22 μm filter. Proteins in the filtered supernatant were concentrated 20× at 4°C, using 3K MWCO Vivaspin 20 Centrifugal Concentrators (Sartorius Corporation, Germany). Protein was quantified using the Bradford assay (Pierce Bradford Protein Assay kit, Thermo Scientific), as per manufacturer's instructions and enzyme assays were then performed with approximately 0.4–0.5 mg mL^−1^ concentrated protein as above.

### 2.7 PolyP Degradation Assay

PolyP degradation was directly measured in cell‐free extracts of *H. volcanii* by silica column extraction (Lee et al. 2018), followed by DAPI quantification (Aschar‐Sobbi et al. 2008; Bru et al. 2017). Enzyme assays were prepared as above, but in 0.4 mL reaction volumes with 5 mM of high purity polyP type 60 (polyP60) with an average chain length of 60 Pi residues (RegeneTiss Incorporated, JP).

Triplicate 50 μL samples were taken at *T*_0_ and *T*_end_ of assays and extraction followed the methods of Lee et al. (2018), using QIAquick spin columns (Qiagen, USA). Elution was repeated twice for a total volume of 180 μL. Eluent was combined with 20 μL of nuclease assay buffer (1 mM CaCl_2_, 10 mM MgCl_2_ and 100 mM Tris–HCl pH 7.4) and treated with DNase I (Thermo Scientific Inc., USA) and RNase A (Thermo Scientific Inc., USA) for 60 min at 37°C. Nucleases were inactivated with 10 μL of 0.5 M EDTA and purified polyP was quantified immediately.

PolyP quantification was based on the methods of Aschar‐Sobbi et al. (2008) and Bru et al. (2017). Assays were performed in triplicate in a black 96‐well microtiter plate. Fifty microlitres of each standard (0 to 80 μM polyP60 in 50 mM Tris–HCl pH 7.4 and 2 mM EDTA) was added to wells, alongside 2–10 μL of extracted polyP made up to 50 μL with 500 mM Tris–HCl pH 7.4. Then, 50 μL of 50 μg mL^−1^ DAPI solution was added and the plate incubated in the dark at RT for 10 min. Fluorescence intensity was measured on a FLUOstar Omega Plate Reader (BMG LABTECH, Germany) at ex/em 415/558 nm.

### 2.8 Zymography

Native‐PAGE was used for in‐gel detection of polyP degradation activity by adapting the methods of McGrath et al. (1995) and Samper‐Martín et al. (2021). Cell‐free protein extracts of *H. volcanii* grown on 1 mM PolyP45 were run on Mini 1 mm Novex WedgeWell Tris‐Glycine 8%–16% gels (Thermo Scientific Inc., USA) with a Novex XCell mini electrophoresis system (Thermo Scientific Inc., USA). Sample loading buffer (2×) consisted of 0.2 M Tris–HCL pH 8.6, 0.2% w/v glycerol and 0.1% w/v bromocresol green, while native running buffer consisted of 25 mM Tris–HCl pH 8.6, 192 mM glycine and 1 mM of polyP45. Before sample addition, 1.5 μL of a mixture of 500 mM polyP45, polyP60 and polyP130 was run on the gel for 5 min at 150 V. Then, 25 μL of cell‐free extract (100–200 ng) was loaded onto gels along with a PageRuler Plus Pre‐stained Protein Ladder (Thermo Scientific Inc., USA) and run at 150 V for 90 min. Gels were rinsed with dH_2_O and incubated in 30 mL of assay buffer (50 mM HEPES, 1 M KCl, 1.25 M NaCl, 10 mM PolyP45 and 2 mM MnCl_2_) for 2 h at 37°C. After rinsing with dH_2_O, lanes were carefully separated for staining and imaging with an Azure 280 gel imager (Azure Biosystems Inc., USA).

Proteins were stained with Coomassie Brilliant Blue solution (0.1% Brilliant Blue, 40% ethanol and 10% acetic acid) for 30–60 min, washed with dH_2_O and de‐stained with a 10% v/v ethanol and 7.5% v/v acetic acid solution for 1–3 h until background staining reduced. Staining for Pi was performed with malachite green (Queirozclaret and Meunier 1993), or with BIOMOL green (Enzo Life Sciences Inc., UK). Gel sections were immersed in malachite green solution (2 mM malachite green, 3 M H_2_SO_4_ and 2% ammonium molybdate) or BIOMOL green solution for up to 1 h, until staining was observed. PolyP staining was carried out by immersing gels in fixing solution (40% v/v ethanol and 10% v/v acetic acid) containing 4 μg mL^−1^ DAPI for 30 min in the dark. Gels were then washed twice with fixing solution without DAPI for 30 min in the dark and then imaged with UV light at 365 nm.

### 2.9 Protein Homology Searches

The annotated reference protein assembly for *H. volcanii* DS2 (GCF_000025685.1) was retrieved from NCBI RefSeq (O'Leary et al. 2015). Amino acid sequences of characterised polyP metabolizing proteins (Table A1) were obtained from the NCBI or UniProtKB protein databases and searched for in the protein assembly using the protein–protein BLAST (BLASTP) program of NCBI BLAST+ v2.9.0 (Camacho et al. 2009). The BLASTP results were restricted to an e‐value below 1 × 10^−10^ and a minimum percentage query coverage of 70%.

### 2.10 Statistical Tests

Quantitative data presented in figures are the mean of three biological replicates and error bars represent the Standard Error of the Mean. Statistical analysis was performed by one‐way or two‐way ANOVA with Turkeys multiple comparison post hoc tests and significance was ascribed to data with *p* values below 0.05.

## 3 Results

### 3.1 Distribution of PolyP Metabolizing Enzymes in *H. volcanii*

Previous work has highlighted that many haloarchaea contain bacterial homologues of polyP synthesizing genes but lack the corresponding genes for its degradation. To uncover potentially novel pathways for the degradation of polyP, the model haloarchaeon *H. volcanii* was selected here for further investigation. Bioinformatic analysis of the *H. volcanii* genome (summarised in Table 1) confirmed that *H. volcanii* encodes homologues of both the bacterial enzymes PPK1 (synthesis of polyP from ATP) and PPK2 (reversible synthesis of polyP from ATP). However, no homologues of bacterial PPXs, the canonical bacterial enzyme able to convert polyP into free Pi, were encoded in the genome.

**TABLE 1:** A comparison of the homologues of potential polyphosphate (polyP) metabolizing enzymes identified in the genome of *Haloferax volcanii* DS2, along with other well‐characterised polyP‐degrading enzymes and the enzyme identified in this study.

	Mechanism of action	Mass (kDa) and accession number of enzyme(s) in *H. volcanii*	Mass (kDa) of prototypical enzyme	Predicted metal cofactors	References
Polyphosphate Kinase 1 (PPK1)	Intracellular phosphorylation of polyP, coupled with dephosphorylation of nucleoside phosphates	83.7 (ADE04455.1) 93.7 (ADE04895.1)	—	Mg^2+^	(Muller et al. 2019)
Polyphosphate Kinase 2 (PPK2)	Intracellular dephosphorylation of terminal residues of polyP, coupled with phosphorylation of nucleoside phosphates	33.1 (ADE03366.1)	—	Mg^2+^	(Neville et al. 2022)
Exopolyphosphatase (PPX)	Intracellular hydrolysis of terminal Pi residues from polyP, releasing free Pi	—	~35 or ~60	Mg^2+^ (> > Mn^2+^, Fe^2+^, Co^2+^)	(Song et al. 2019)
Alkaline Phosphatase (AP)	Extracellular hydrolysis of terminal Pi residues from polyP, releasing free Pi	—	~81–89	Mg^2+^ > Zn^2+^	(Zakataeva 2021)
Survival protein E (SurE)	Intracellular hydrolysis of terminal Pi residues from polyP, releasing free Pi	27.8 (ADE05110.1) 31.5 (ADE01842.2)	—	Mg^2+^ (> > Mn^2+^, Ni^2+^, Co^2+^)	(Zakataeva 2021)
NAD kinase (NADK)	Intracellular dephosphorylation of terminal residues of polyP, coupled with the phosphorylation of NAD^+^	27.7 (ADE02743.1)	—	Mg^2+^ / Mn^2+^ / Ca^2+^ / Zn^2+^ / Co^2+^	(Sakuraba et al. 2005)
Polyphosphate‐degrading enzyme identified in this study	Intracellular hydrolysis of terminal Pi residues from polyP, releasing free Pi	~45	—	Mn^2+^ > Co^2+^	—

Furthermore, *H. volcanii* also did not encode any homologues of common intracellular or extracellular acid/alkaline phosphatases—enzymes capable of dephosphorylating organic phosphorus compounds and sometimes also short‐chain polyP. Genes encoding the characterised eukaryotic polyP metabolizing enzymes were also absent in *H. volcanii* . However, the *H. volcanii* genome does contain homologues of a SurE 5′ nucleotidase and an NADK enzyme (Table 1). While polyP is not usually catabolised by these enzymes, certain homologues have been previously reported to degrade short‐chain polyP alongside their primary substrates (Proudfoot et al. 2004; Sakuraba et al. 2005; Zakataeva 2021). We thus sought to determine if *H. volcanii* can degrade polyP, and if so, the enzymes responsible and whether they represent a novel pathway for polyP catabolism.

### 3.2 Growth of *H. volcanii* on Exogenous PolyP

PolyP metabolism in *H. volcanii* was first investigated by testing whether this organism could consume and thus degrade extracellular polyP, utilizing the biopolymer as its sole source of phosphorus for growth as some other environmental microorganisms have been shown to do. For this, cultures of *H. volcanii* were first starved of all phosphorus and then incubated with either 1 mM of polyP or Pi as their sole phosphorus source or no phosphorus as a negative control.

**FIGURE 2:**
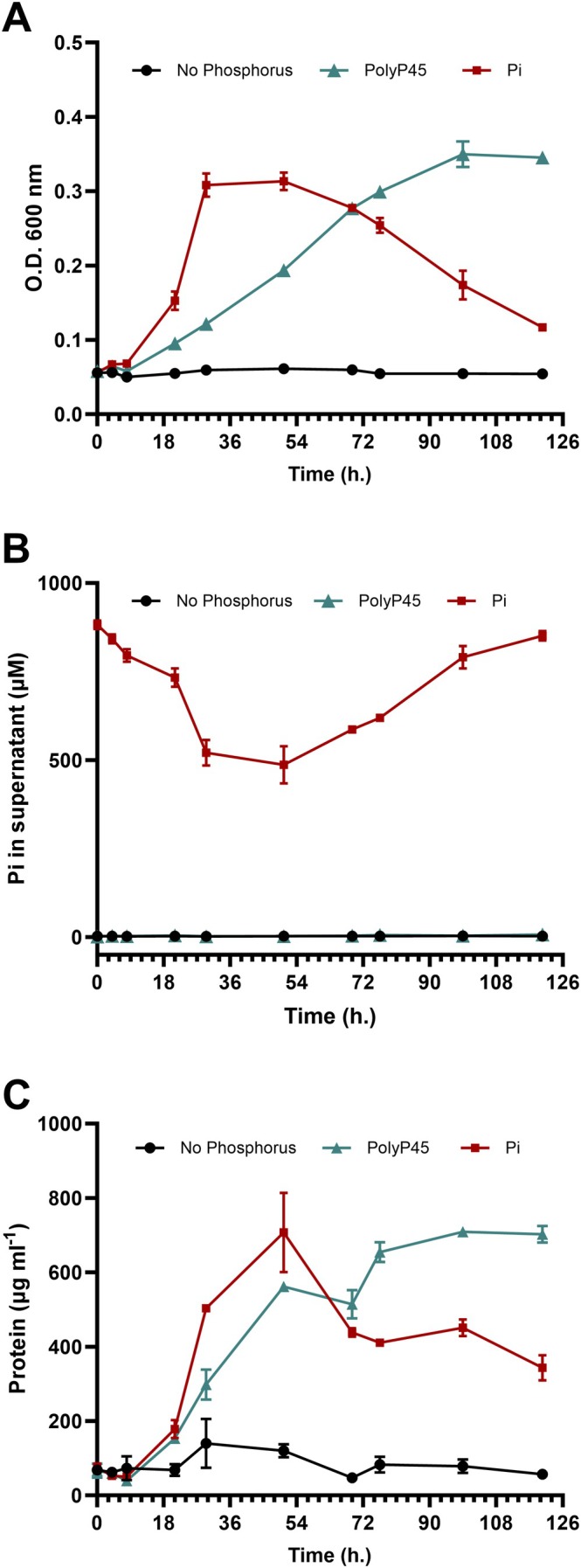
A growth curve of *Haloferax volcanii* with inorganic polyphosphate type 45 (PolyP45) as the sole phosphorus source (cyan triangles), versus growth on phosphate (red squares) or no phosphorus (black circles). Growth is displayed as the optical density at 600 nm (A) and protein concentration (B). Exogenous inorganic orthophosphate (Pi) concentration in culture medium was also measured throughout (C). The error bars display the standard error of the mean of three biological replicates.

Cells of *H. volcanii* grew well with exogenous polyP as the sole source of phosphorus. Cultures grown on polyP reached a maximum O.D. 600 nm of ~0.35 after 99 h, compared to the cultures grown on Pi that reached a maximum O.D. 600 nm of ~0.31 at 50 h (Figure 2A). The total protein concentration in cultures was also monitored as a further proxy for growth, as O.D. readings of pigmented haloarchaeal cultures can be inaccurate. Total protein in polyP and Pi grown cultures reached comparable concentrations after 50 and 99 h respectively (Figure 2B). Therefore, polyP appears to support comparable growth of *H. volcanii* cells as with Pi, while the slower growth rate in cultures grown on polyP could likely be expected when growing an organism on a new, unconventional substrate.

No growth occurred in phosphorus‐free control cultures, confirming that *H. volcanii* cells were sufficiently starved of phosphorus prior to experimentation (Figure 2A). Therefore, the observed growth could be fully attributed to the phosphorus source being tested (Pi or polyP). The Pi concentration in culture supernatants was also monitored, confirming that no contaminating Pi was present from the abiotic degradation of polyP or external sources (Figure 2C).

### 3.3 Detection, Localisation and Regulation of PolyP‐Degrading Activity

**FIGURE A1:**
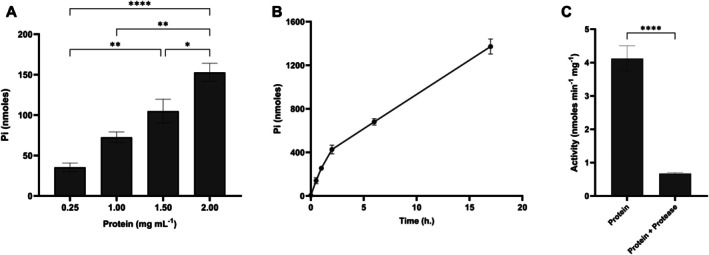
The relationship between inorganic phosphate (Pi) release from polyphosphate (type 45; polyP45) and protein concentration (A), or time (B), in enzyme assays performed with cell‐free extracts of *Haloferax volcanii* grown on polyP45 (1 mM) as the sole phosphorus source. Additionally, the effect of protease treatment on cell‐free extract prior to assaying is shown (C). Error bars indicate standard error of the mean where *n* = 3. Asterisks indicate statistical significance, where *p* < 0.05 (*), *p* < 0.01 (**), *p* < 0.0001 (****).

The capacity to degrade polyP in *H. volcanii* was investigated with enzyme assays using cell‐free extracts prepared from *H. volcanii* grown on polyP as the sole phosphorus source. A polyP‐degrading activity was subsequently confirmed in these assays of cell‐free extracts of *H. volcanii* by adding polyP as the sole substrate and observing its conversion to free Pi. This appears consistent with a PPX‐type mechanism (involving the cleavage of terminal Pi residues; Song et al. 2019) despite no such homologue being encoded by *H. volcanii* , suggesting that an unknown polyP‐degrading enzyme is responsible for this activity. PolyP‐degrading activity also increased with increasing protein concentration, decreased with time and the addition of protease significantly inhibited activity, confirming that this was enzymatic and not some abiotic/chemical phenomena (Figure A1).

**FIGURE 3:**
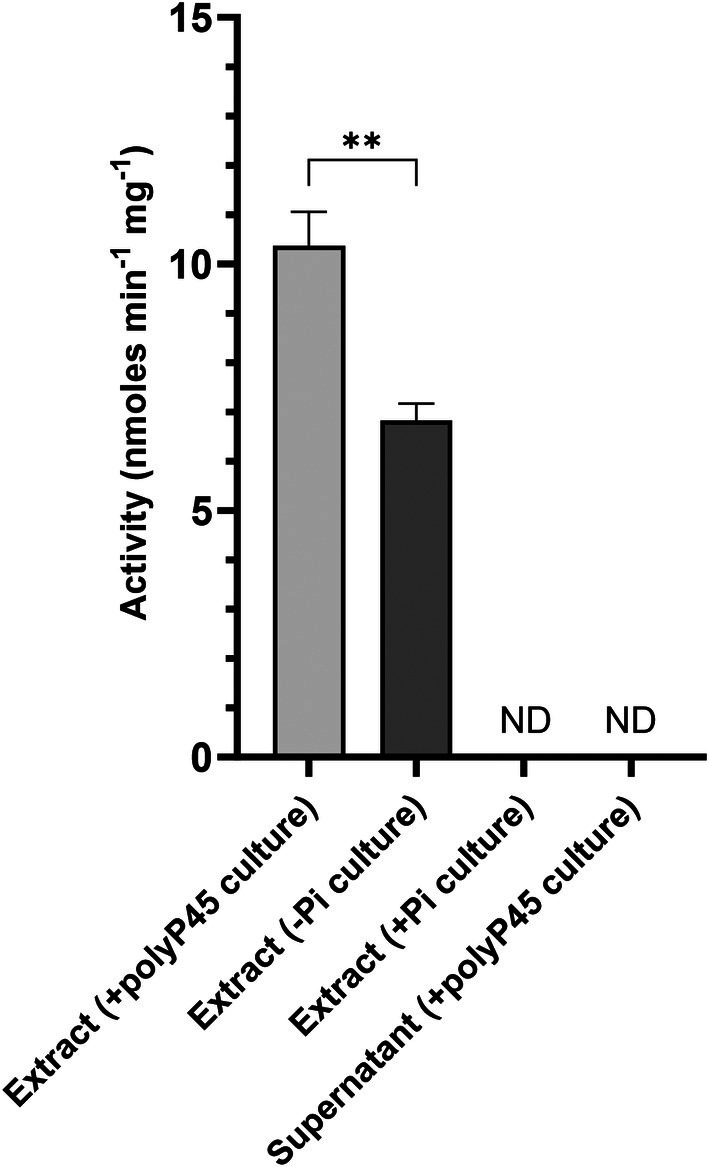
Polyphosphate (type 45; polyP45) degrading activity in culture supernatant and cell‐free extracts of *Haloferax volcanii* cells grown on polyP45 (1 mM) as the sole phosphorus source. Also shown is activity from extracts grown on inorganic orthophosphate (Pi; 1 mM) or with no phosphorus source at all (−Pi). ND denotes no activity was detectable. Error bars indicate standard error of the mean where *n* = 3. Asterisks indicate statistical significance, where *p* < 0.01 (**).

The concentrated supernatant of *H. volcanii* cells grown on polyP was also found to have no detectable polyP‐degrading activity (Figure 3), indicating that the enzyme(s) responsible for this activity are not excreted into the extracellular medium. Therefore, as activity was only detected in cell‐free extracts recovered from the clarified (soluble) fraction of lysed cells after centrifugation, this suggests that activity is likely mediated by an intracellular, soluble enzyme. However, the possibility that membrane‐associated proteins are solubilised during extraction or that small membrane fragments might remain cannot be excluded with this method.

Enzymatic degradation of polyP was only detected in extracts of *H. volcanii* cells that had been either starved of Pi or starved of Pi and subsequently grown on polyP. Higher activity was observed in extracts from cells grown with polyP than those only starved of Pi (Figure 3). Thus, the catabolism of polyP appears regulated by Pi starvation and the presence of polyP, which is consistent with how the degradation of other phosphorus sources, including organophosphates and organophosphonates, is regulated (McGrath et al. 2013; Santos‐Beneit 2015). This, along with the lack of any enzymatic activity in cell‐free extracts of cells grown solely on Pi (Figure 3), illustrates that the polyP‐degrading enzyme activity observed here is responsible for the ability of *H. volcanii* to grow on polyP as its sole source of phosphorus.

PolyP degradation was also measured directly by monitoring the disappearance of the polyP substrate in the same polyP‐containing enzyme assays with cell‐free extracts of *H. volcanii* cells grown on polyP. Using this approach, the concentration of polyP showed a reduction of ~354 nmol polyP (as Pi equivalent) over 16 h.

### 3.4 Characterisation of PolyP‐Degrading Activity

#### 3.4.1 Halophilicity

**FIGURE 4:**
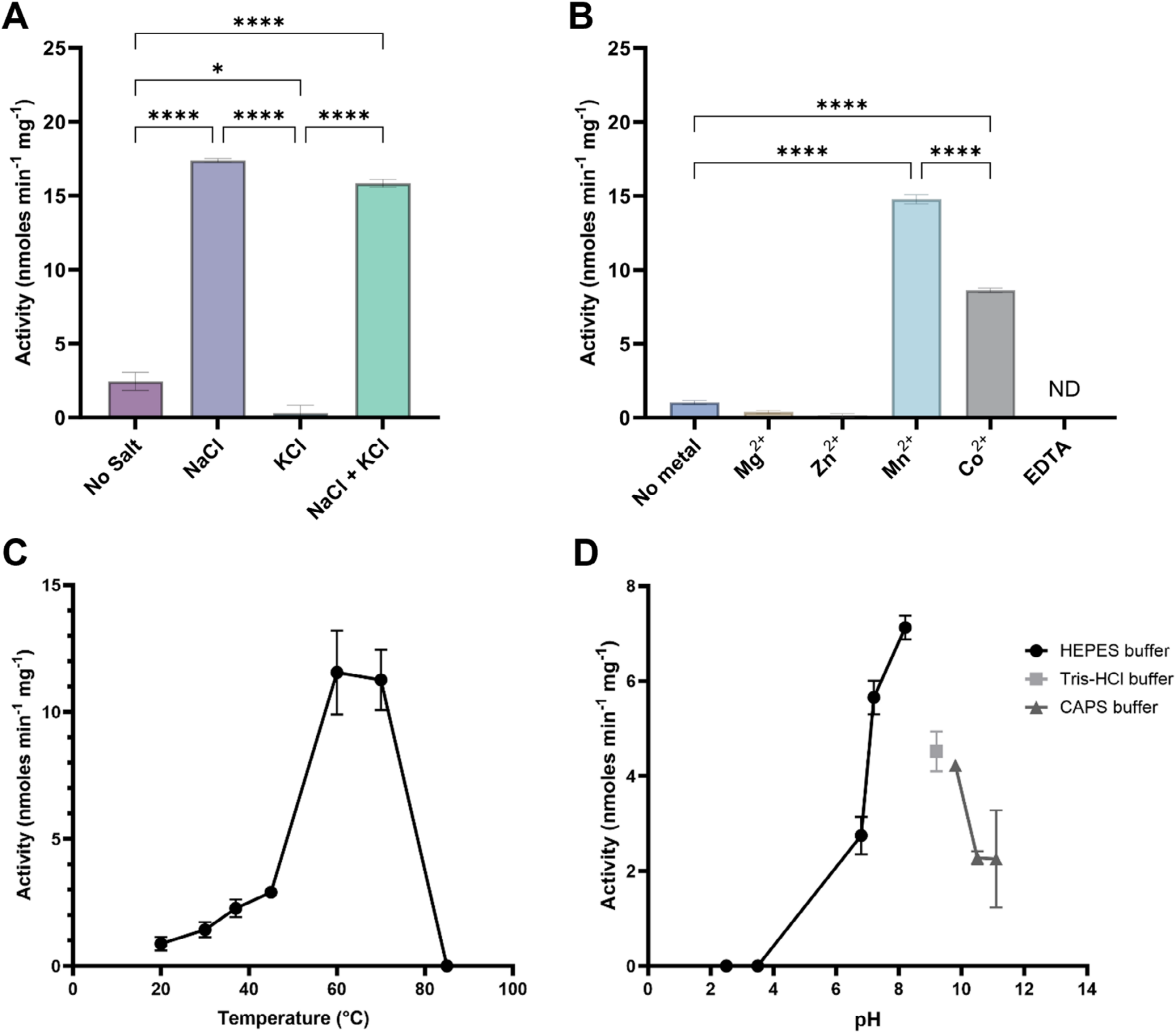
The effect of 1.25 M sodium chloride (NaCl) and 1 M potassium chloride (KCl) salts (A), EDTA and various metal ion cofactors (B), temperature (C) and pH (D) on the polyphosphate (type 45; polyP45) degrading enzyme activity in cell‐free extracts of *Haloferax volcanii* grown on polyP45 (1 mM). Metal ions were at a concentration of 1 mM and EDTA was at 10 mM. Error bars indicate standard error of the mean where *n* = 3. Asterisks indicate statistical significance, where *p* < 0.05 (*) and *p* < 0.0001 (****).

Haloarchaeal enzymes are specially adapted to be active in high‐salt conditions that inhibit most other enzymes (Dassarma and Dassarma 2015). To determine whether this polyP‐degrading enzymatic activity was halophilic, the effect of adding NaCl and/or KCl to enzyme assays was tested. NaCl and KCl were only tested at one concentration because the aim was solely to determine whether salts enhanced activity. The polyP‐degrading activity in *H. volcanii* was found to be halophilic, exhibiting a marked increase in activity with 1.25 M NaCl compared to in assays with no added salts (Figure 4A). Notably, ~15% of maximal activity persisted under salt‐free conditions and adding 1 M KCl, alone or in addition to 1.25 M NaCl, did not enhance activity (Figure 4A).

Given haloarchaea accumulate high intracellular K^+^ (up to 4–5 M) to counter osmotic stress, a preference for Na^+^ might imply the enzymatic activity is located in the outer membrane where high NaCl concentrations might be encountered. But many cytosolic haloarchaeal enzymes also exhibit distinct ion preferences for structural stability or activation (Madern et al. 2000). Furthermore, the lack of inhibition with KCl also suggests that the enzyme could remain active in the intracellular environment. Future investigation of the exact effects of salts on the enzyme/s responsible for this activity is required, which may reveal more about the localisation and enzymatic mechanisms behind this activity.

#### 3.4.2 Metal Cofactor Requirements

The polyP‐degrading activity was metal‐dependent, showing strong activation by Mn^2+^ and Co^2+^ (Mn^2+^ > Co^2+^) and complete inhibition by EDTA (Figure 4B). At 1 mM, Mn^2+^ increased activity 15‐fold, while Co^2+^ produced an 8‐fold increase. The significantly reduced activity observed without metal ions likely is the result of the use of EDTA during cell‐free extract preparation, which would chelate most of the metal ions present in the original extract or loosely bound to enzymes.

*H. volcanii* only encodes one homologue of a characterised polyP‐degrading enzyme, PPK2, which requires Mg^2+^ as an essential co‐factor (Neville et al. 2022). Therefore, the absence of activity with Mg^2+^ appears to negate PPK2 being responsible for the polyP‐degrading activity observed here. Other non‐specific enzymes that could potentially degrade polyP (SurE and NADK, see Table 1) are also encoded by *H. volcanii;* yet these enzymes are also primarily activated by Mg^2+^.

#### 3.4.3 Temperature and pH

The polyP‐degrading activity was also moderately alkaliphilic and thermophilic, with an optimum pH of 8.2 and a temperature of 60°C (Figure 4C,D). These characteristics are typical of haloarchaeal enzymes due to the hypersaline environments inhabited by these organisms that frequently experience temperature fluctuations and variable pH (Martínez et al. 2022).

#### 3.4.4 Substrate Range

**FIGURE 5:**
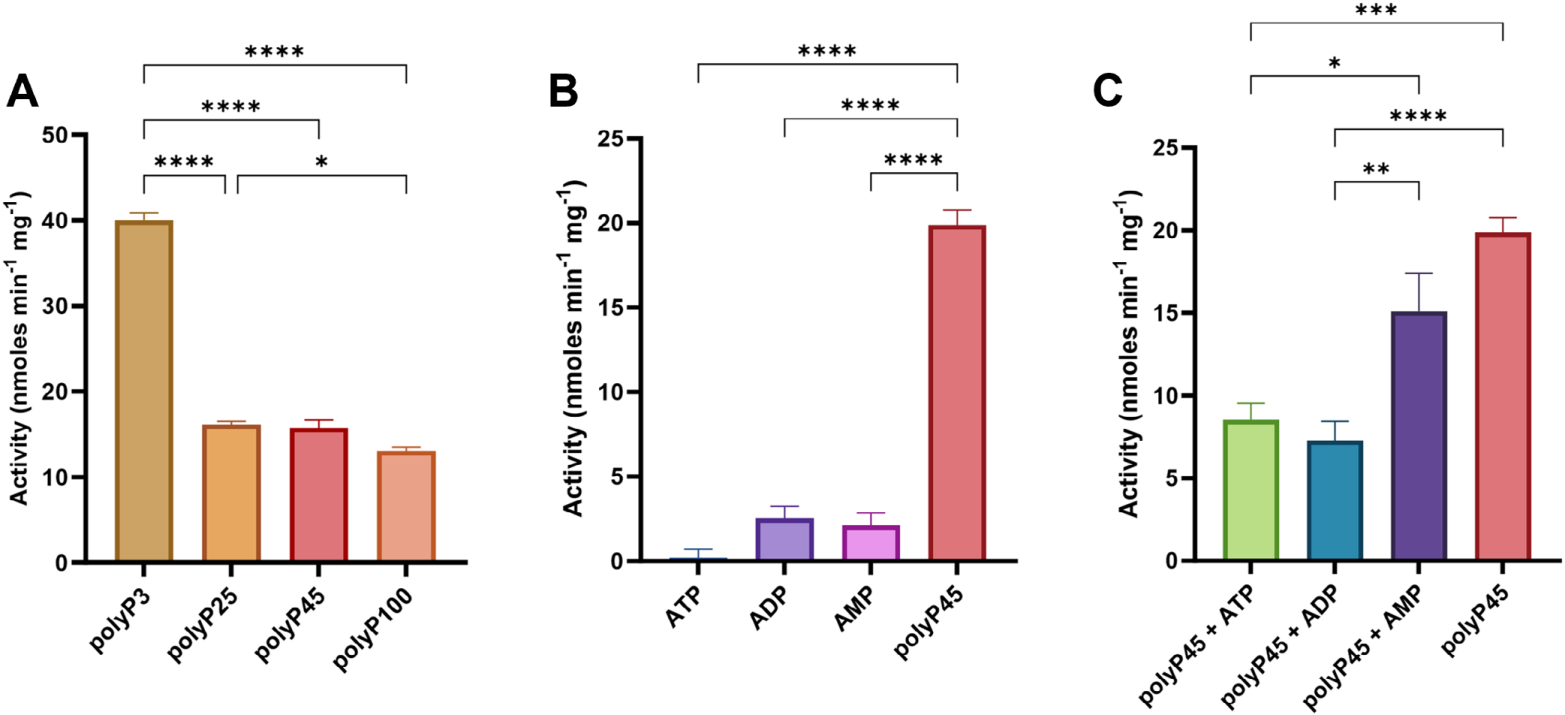
The ability to degrade various lengths of polyphosphate (A) or nucleoside phosphates (B) in cell‐free extracts of *Haloferax volcanii* grown on polyphosphate (type 45, PolyP45; 1 mM) is shown, along with inhibitory effects of nucleoside phosphates on polyphosphate (PolyP45) degrading activity (C). Various polyphosphate polymers (tripolyphosphate, polyP3; polyphosphate type 25, polyP25; polyP45; polyphosphate type 100, polyP100) and adenosine mono‐, di‐ and tri‐phosphate compounds (ATP, ADP and AMP) were tested at 10 mM. Error bars indicate standard error of the mean where *n* = 3. Asterisks indicate statistical significance, where *p* < 0.05 (*), *p* < 0.01 (**), *p* < 0.001 (***) and *p* < 0.0001 (****).

PolyP degradation was also observed across a range of polyP polymer sizes, spanning chain lengths from 3 to ~100 Pi residues (Figure 5A). Enzyme activity was highest with PolyP3 as substrate, the shortest polymer tested, being ~2.5‐fold higher than that observed with the longer polyP polymers (Figure 5A). Both PolyP25 and PolyP45 polymers exhibited similar rates of activity at approximately 16 nmoles min^−1^ mg^−1^, while activity with PolyP100 was lower at 13.8 nmoles min^−1^ mg^−1^ (Figure 5A).

Although higher activity was observed with short‐chain polyP consisting of three Pi residues (Figure 5A), interpretation of substrate preference is complicated here by the use of crude cell‐free extract assays. These extracts may also contain other non‐specific enzymes (e.g., phosphatases or kinases) capable of acting on short‐chain polyP (Malinen et al. 2007; Kajander et al. 2013; Moeder et al. 2013). Therefore, it remains unclear whether degradation of short‐ and long‐chain polyP is mediated by the same enzyme(s).

Importantly, degradation of longer‐chain polyP polymers (25–100 Pi residues) was consistently observed under these conditions. As the degradation of long‐chain polyP and release of Pi is typically associated with specialised polyP‐degrading enzymes and the only potential candidate encoded by *H. volcanii* (PPK2) requires Mg^2+^ for activity and does not catalyse the release of free Pi, this observation supports the presence of a previously unknown polyP‐degrading activity in *H. volcanii* .

Nucleoside phosphates are also common alternate substrates for many polyP‐degrading enzymes (Albi and Serrano 2016). However, the addition of either 10 mM of ATP, ADP, or AMP to cell‐free extracts of *H. volcanii* cells grown on polyP resulted in minimal observed activity (Figure 5B). Conversely, when ATP or ADP were added to assays along with polyP, activity was reduced by ~43% and ~37% respectively (Figure 5C). This might suggest that nucleoside phosphates can interact with the reaction mechanism of polyP degradation observed here. However, due to the use of crude cell‐free extracts, it is also possible that ATP/ADP stimulate other enzymes in the extract to consume the Pi released from polyP, giving the appearance of reduced polyP‐degrading activity.

### 3.5 Zymography of the PolyP‐Degrading Enzyme

**FIGURE 6:**
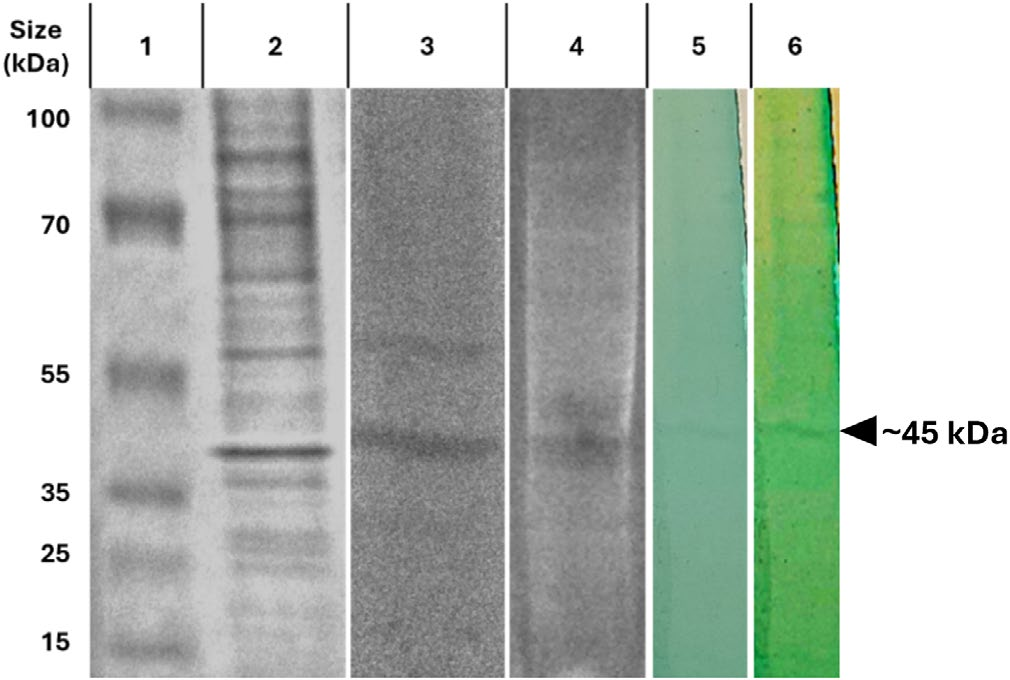
Zymograms of polyphosphate degradation in cell‐free extracts of *Haloferax volcanii* grown on polyphosphate type 45 (1 mM). A protein standard (Lane 1) and cell‐free extract (Lane 2) were stained with Coomassie. Lanes 3, 4 and 5 contained cell‐free extract and were subsequently subjected to assay incubations. Lane 3 was stained with malachite green, Lane 4 with DAPI and Lane 5 with BIOMOL green. Lane 6 is a pseudo‐coloured copy of Lane 5. Inorganic phosphate (Pi) release is indicated by dark bands in Lanes 3, 5 and 6. Absence of polyphosphate is indicated by a broad dark band in Lane 4.

To complement the biochemical characterisation of this novel polyP‐degrading enzyme activity, an estimation of the apparent molecular mass of the associated native protein was undertaken using zymography. With this approach, crude cell‐free extract is first separated on a native polyacrylamide gel preloaded with the polyP substrate. In‐gel activity assays are then performed and subsequent staining for either Pi release or polyP degradation localises the polyP‐degrading activity to a protein band on the gel (Figure 6).

Staining for Pi release (with BIOMOL or malachite green) resulted in the appearance of bands at an apparent molecular mass of ~45 kDa (Lanes 3, 5 and 6, Figure 6). Staining for polyP using DAPI also revealed a corresponding region of polyP loss at approximately the same position (Lane 4, Figure 6). Therefore, the co‐occurrence of Pi release and polyP disappearance at this same position indicates that a protein migrating at an apparent molecular mass of ~45 kDa is associated with the observed polyP‐degrading activity. However, as zymography provides only an approximation of the native mass under non‐denaturing conditions, the oligomeric state and the precise molecular weight remain undetermined. Furthermore, haloarchaeal proteins are enriched in acidic residues, which affect how they migrate during gel electrophoresis and potentially cause them to run faster through the gel.

Nevertheless, the co‐localisation of polyP degradation and Pi release to a discrete band with zymography supports the presence of a specific enzyme associated with this novel polyP‐degrading activity. While the molecular weight should be considered an approximate estimate, it still differs significantly from the molecular weights of previously characterised polyP enzymes encoded by *H. volcanii* that could be responsible for such polyP‐degrading activity (i.e., PPK2, SurE, NADK, see Table 1). Taken together with the distinct biochemical properties described above, these findings strongly support the presence of a previously unrecognised polyP‐degrading enzyme in *H. volcanii* .

## 4 Discussion

Our investigations of exogenous polyP consumption by *H. volcanii* provide evidence for a novel enzymatic pathway for polyP degradation, whereby polyP appears to be imported and degraded intracellularly as a source of phosphorus for growth. The enzyme associated with this activity is halophilic, requires Mn^2+^ or Co^2+^, liberates Pi from polyP, and appears likely to be cytosolic. These properties distinguish this enzyme from those potentially capable of degrading polyP in *H. volcanii* and from previously characterised polyP‐degrading enzymes (Table 1). While this broadens our understanding of archaeal polyP metabolism and the bioavailability of polyP, further studies including purification, genetic and structural analyses are required to fully characterise this enzymatic pathway.

A key unresolved question arising from these findings is how extracellular polyP is accessed and transported into the cell prior to degradation. The absence of activity and lack of Pi release in the extracellular medium is consistent with an intracellular activity. Additionally, activity in cell‐free extracts and its localization to a discrete band during native‐PAGE further suggests the associated enzyme is soluble. However, we cannot exclude the possibility that membrane‐associated or periplasmic components contribute to the observed activity and further work is required to fully elucidate the cellular location.

If polyP degradation in *H. volcanii* is indeed cytosolic, then the intracellular degradation of polyP contrasts with typical microbial processing of other complex phosphorus compounds—including those structurally similar to polyP such as nucleoside phosphates and eDNA. These compounds are usually dephosphorylated by secreted or membrane‐bound acid/alkaline phosphatases and the liberated Pi is subsequently imported. While here the mechanism of polyP import into *H. volcanii* cells prior to degradation remains unknown, many haloarchaea (including *H. volcanii* ) contain machinery for the uptake of eDNA which may provide a functional analogy for their import (Chimileski et al. 2014; Oren 2014; Wagner et al. 2017; Chen et al. 2019). Identifying the mechanisms responsible for polyP import would be important to further investigate the diversity, distribution and evolutionary history of this metabolic trait.

The ability of *H. volcanii* to consume exogenous polyP when Pi is not available may not be surprising as haloarchaea are adapted to hypersaline environments that are often oligotrophic and starved of Pi (Oren 1983; Atkinson 1987). Many microorganisms found in other oligotrophic environments have evolved similar adaptations to consume alternative phosphorus sources such as organophosphates or organophosphonates instead of Pi (Orchard et al. 2010; McGrath et al. 2013; Filella et al. 2022). Additionally, the polypoid nature of *H. volcanii* has been suggested to be an example of one such adaptation, with excess DNA potentially acting as a nutrient store (Zerulla et al. 2014). Given that some haloarchaea are involved in denitrification, ammonia oxidation and sulfur reduction (Bonete et al. 2008; Oren 2016; Sorokin et al. 2016), the potential for polyP to act as a reservoir of bioavailable phosphorus suggests that Pi‐limitation can be overcome and key microbial processes sustained in these ecosystems. Thus, the potential bioavailability of polyP could affect not only the cycling of phosphorus but also impact coupled biogeochemical cycles, including carbon, nitrogen and sulfur cycling, in hypersaline environments.

Many moderate environments are also Pi‐limited due to the rapid cycling and relatively low environmental concentrations of Pi, which is considered a significant constraint on biological productivity. While few attempts have been made to quantify dissolved extracellular polyP in the environment, microbial polyP can constitute up to ~30% of total biological phosphorus, and the constant lytic turnover of these microbial would thus suggest that polyP could contribute to bioavailable phosphorus pools in some environments (Shinohara et al. 2012; Martin et al. 2014, 2018; Yang et al. 2024). Thus, when taken together with studies showing that some marine microorganisms cycle extracellular polyP, this further suggests that polyP could play an underappreciated role in the global biogeochemical phosphorus cycle (Diaz et al. 2019; Duhamel et al. 2021; Filella et al. 2022).

PolyP consumption has been observed in certain marine microorganisms including diatoms (Diaz et al. 2019), phytoplankton (Diaz et al. 2018), cyanobacteria (Filella et al. 2022), and the bacterium *Ruegeria pomeroyi* (Adams et al. 2022). While most did not propose a mechanism, some studies proposed that extracellular acid/alkaline phosphatases mediated the degradation of short‐ to medium‐chain polyP (3–45 monomers) (Huang et al. 2018; Martin et al. 2018; Adams et al. 2022), but there is no direct biochemical evidence for this. These putative mechanisms also differ substantially to the novel pathway identified here, highlighting that much remains unknown about the diversity of polyP consumption mechanisms and how widespread these could be.

Overall, this study provides biochemical evidence for a previously unrecognised archaeal polyP‐degrading enzyme. This work furthers a very limited understanding of archaeal polyP metabolism, highlighting that much remains to be discovered in the archaea. It also lays a foundation for investigating polyP as a bioavailable constituent of DIP pools and the microbial processes that govern polyP cycling. As phosphorus availability directly shapes microbial community dynamics and influences coupled biogeochemical cycles, unravelling the bioavailability of diverse phosphorus species is important to understanding the impacts microorganisms have on broader biogeochemical fluxes (Duhamel et al. 2021). Further characterisation of this novel polyP‐degrading enzyme and the exact mechanism of polyP uptake and degradation will be important to understanding the prevalence and ecological relevance of this metabolic trait across environments and microbial communities.

## Author Contributions

**Jack W. F. Nicholls:** conceptualization, investigation, writing – original draft, visualization, writing – review and editing. **John W. McGrath:** conceptualization, funding acquisition, writing – original draft, writing – review and editing, supervision, investigation. **Jason P. Chin:** conceptualization, funding acquisition, writing – original draft, writing – review and editing, supervision, investigation. **Timothy M. Lenton:** conceptualization, funding acquisition, writing – review and editing, supervision. **Vincent O'Flaherty:** conceptualization, funding acquisition, writing – review and editing, supervision.

## Funding

This work was supported by the Department for the Economy; Biotechnology and Biological Sciences Research Council, BB/W019531/1.

## Conflicts of Interest

The authors declare no conflicts of interest.

## Data Availability

The data that support the findings of this study are available on request from the corresponding author.

## Appendix A

**TABLE A1:** Characterised enzymes known to be involved in the microbial cycling of polyphosphate that were used to search for homologues in the *Haloferax volcanii* genome.

Function	Enzyme	Enzyme name	Gene	Organism	NCBI accession	UniProt accession	Taxonomy
Polyphosphate synthesis	PPK1	Polyphosphate kinase 1	*ppk1*	*Helicobacter pylori*	WP_001078748.1		Bacteria and Archaea
				*Escherichia coli*	WP_000529576.1		
				*Vibrio cholerae*	WP_001271572.1		
				*Methanosarcina mazei*		A0A4P8R194	
	VTC4	Vacuolar transport chaperone 4	*vtc4*	*Saccharomyces cerevisiae*		P47075	Eukarya
				*Trypanosoma cruzi*		W0IC88	
	*Dd*PPK1	*Dictyostelium discoideum* polyphosphate kinase 1	*ppkA*	*Dictyostelium discoideum*	XP_629002.1		Eukarya
	*Dd*PPK2	*Dictyostelium discoideum* polyphosphate kinase complex 2	*arpA*	*Dictyostelium discoideum*		Q54I79	Eukarya
			*arpB*			O96621	
			*arpX*			Q54HE7	
Polyphosphate degradation	PPX	Exopolyphosphatase	*ppx*	*Escherichia coli*		P0AFL6	Bacteria and Archaea
				*Aquifex aeolicus*		O67040	
				*Saccharolobus solfataricus*		Q7LWG1	
	PPX‐GPPA	Guanosine‐5′‐triphosphate, 3′‐diphosphate pyrophosphatase	*gppA*	*Escherichia coli*		P25552	Bacteria
				*Helicobacter pylori*	WP_001074178.1		
	PPN1	Endopolyphosphatase 1	*ppn1*	*Saccharomyces cerevisiae*		Q04119	Eukarya
	PPN2	Endopolyphosphatase 2	*ppn2*	*Saccharomyces cerevisiae*		P40152	Eukarya
	DPP1	Diphosphoinositol polyphosphate phosphohydrolase	*ddp1*	*Saccharomyces cerevisiae*		Q99321	Eukarya
	*Sc*PPX1	Yeast exopolyphosphatase	*ppx1*	*Saccharomyces cerevisiae*	NP_012071.1		Eukarya
	TPP	Tripolyphosphatase	*NE1496*	*Nitrosomonas europaea*		Q82UI9	Bacteria and Eukarya
			*TTM3*	*Arabidopsis thaliana*		Q9SIY3	
	*Tb*NH2	NUDIX hydrolase	*TbNH2*	*Trypanosoma brucei*		Q57Z14	Eukarya
	*Tb*NH4		*TbNH4*	*Trypanosoma brucei*		Q583R7	
	PPK2	Polyphosphate kinase 2	*ppk2*	*Ostreococcus tauri*	XP_003080129.1		Bacteria and Archaea
				*Vibrio cholerae*	WP_001086042.1		
				*Meiothermus ruber*		A0A806DL21	
				*Pseudomonas aeruginosa*	WP_003112634.1		
			*pap*	*Acinetobacter johnsonii*		Q83XD3	
				*Pseudomonas aeruginosa*		Q9HYF1	
			*ppk3*	*Ruegeria pomeroyi*		Q5LSN8	
Organic phosphate utilization	PafA	Alkaline phosphatase	*pafA*	*Elizabethkingia meningoseptica*		Q9KJX5	Bacteria and Eukarya
	PhoD		*phoD*	*Bacillus subtilis*	NP_388144.2		
	PhoX		*phoX*	*Chlamydomonas reinhardtii*	XP_001703098.1		
	PhoA		*phoA*	*Escherichia coli*	NP_414917.2		
	PhoC	Acid phosphatase	*phoC*	*Morganella morganii*		P28581	Bacteria
	AphA		*aphA*	*Salmonella typhimurium*		Q540U1	
	OlpA		*olpA*	*Elizabethkingia meningoseptica*		O08351	
	SurE	5′‐nucleotidase (Survival Protein E)	*surE*	*Escherichia coli*		P0A840	Bacteria and Archaea
			*surE*	*Thermotoga maritima*		P96112	
			*surE1*	*Pyrobaculum aerophilum*		Q8ZU79	
			*surE*	*Salmonella typhimurium*		P66881	
	HAP	Phytase	*phyA*	*Aspergillus niger*		P34752	Bacteria and Eukarya
			*appA*	*Escherichia coli*		P07102	
	BPP		*phyC*	*Bacillus subtilis*		O31097	
	PAP		*PAP15*	*Arabidopsis thaliana*		Q9SFU3	
	PTP		*phyA*	*Selenomonas ruminantium*		Q7WUJ1	
